# Mycosis Fungoides Presenting With Multiple Tumors on the Face

**DOI:** 10.7759/cureus.61164

**Published:** 2024-05-27

**Authors:** Naoki Sasaki, Yoko Akamatsu, Akane Ogaya, Tomoko Oda, Shun Ohmori, Etsuko Okada, Yu Sawada

**Affiliations:** 1 Dermatology, University of Occupational and Environmental Health, Kitakyushu, JPN; 2 Dermatology, Japan Community Health Care Organization Shimonoseki Medical Center, Shimonoseki, JPN

**Keywords:** oral therapy, electron beam therapy, bexarotene, face, mycosis fungoides

## Abstract

An 84-year-old female experienced progressive erythema on her limbs and chest over the past year. Initially managed with topical steroids, the erythema eventually spread throughout her body, forming erosions. A biopsy confirmed the diagnosis of mycosis fungoides (MF) (Stage IIB, T2bN0M0B0). Treatment with oral bexarotene (300 mg/day) and narrow-band UVB therapy showed limited improvement. Electron beam therapy (30 Gy in 10 fractions) applied to facial and plantar tumors resulted in a reduction of the tumors. This case highlights the treatment of tumors of MF on the face showing the effectiveness of combining electron beam therapy with bexarotene.

## Introduction

Cutaneous lymphomas primarily consist of T-cell lymphomas, including mycosis fungoides (MF). In the tumor phase, the five-year survival rate falls below 50%, indicating a poor prognosis [1]. Additionally, in cases of tumors developing on the face, they cause significant pain and can make it difficult to open the eyes, substantially impacting quality of life (QOL) [2]. Treatment options such as chemotherapy, phototherapy, and electron beam therapy are typically considered [3]. However, those cases are challenging to improve the QOL by the current therapeutic options. We report a case of MF with extensive tumorous lesions on the face, which showed improvement through a combined treatment of oral bexarotene and electron beam therapy.

## Case presentation

An 84-year-old female initially presented a year ago with erythema on her face and chest, which resolved following treatment with topical steroid ointment. Symptoms relapsed a month before her first consultation at our hospital. At the first visit, extensive infiltrative erythema was observed on the trunk and limbs with multiple nodules on the face accompanied by ulcers and significant pain. She had been undergoing medication treatment for hypertension since the age of 70.

**Figure 1:**
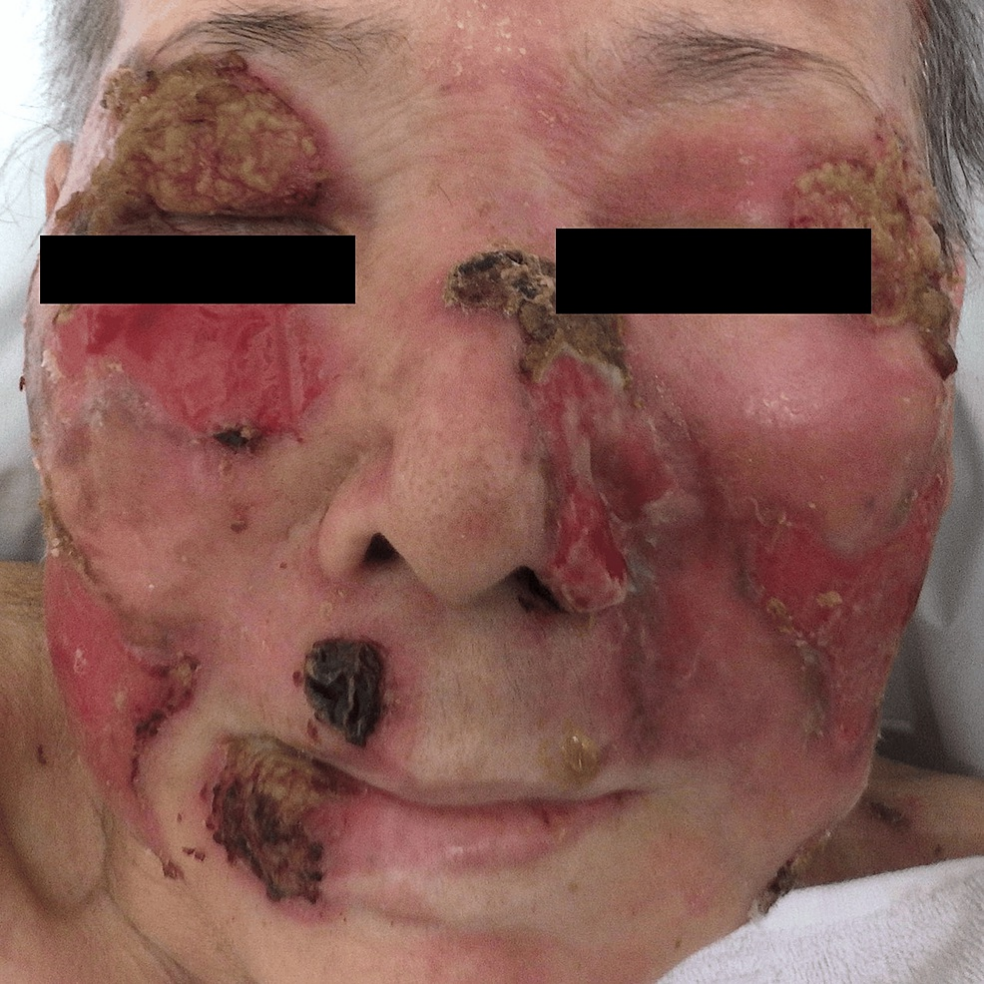
Clinical manifestation Erosion and ulceration with tumors were located on her face.

**Figure 2:**
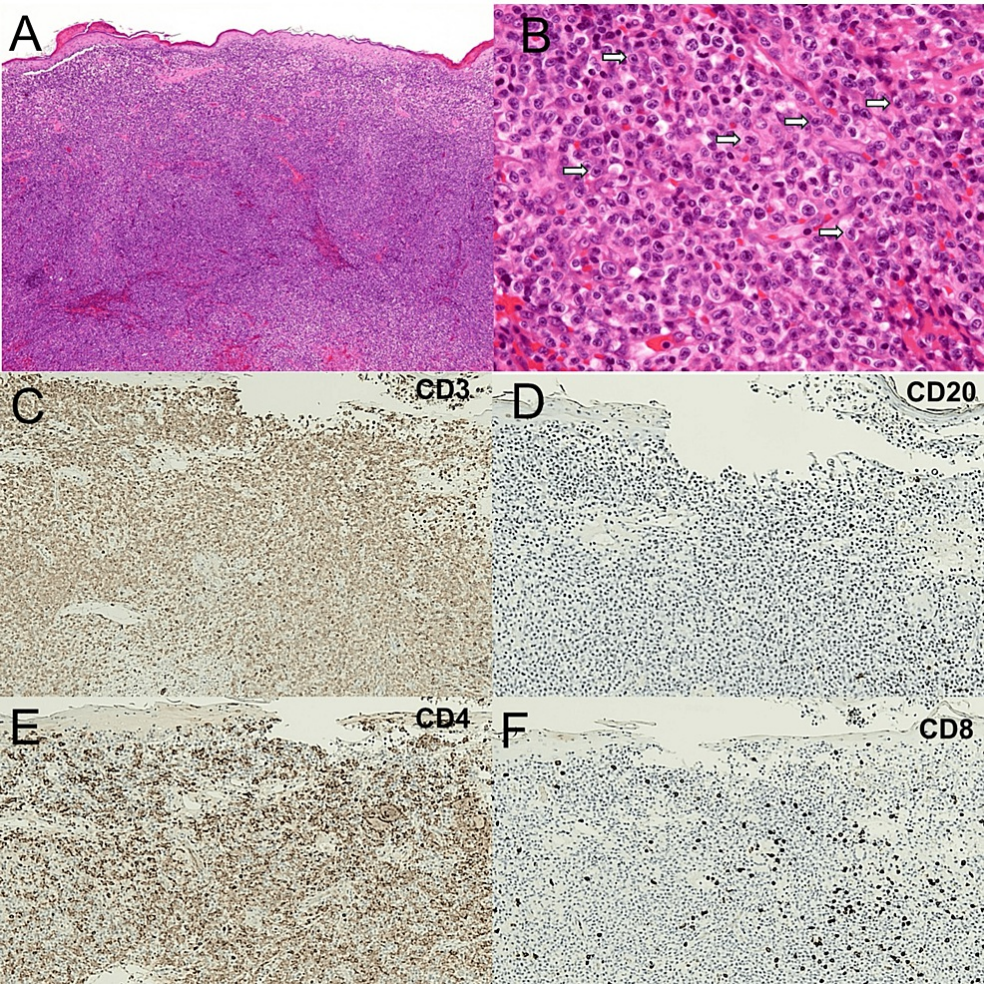
Histological examination A skin biopsy taken from the tumor showed that the infiltrating atypical lymphocytes (Indicated with the white arrow in (B)) were medium-sized with irregular nuclei and predominantly CD4-positive and CD8-negative (A-F). Immunostaining for CD3 (C), CD20 (D), CD4 (E), and CD8 by DAB (diaminobenzidine) staining (F).

Plain computed tomography (CT) imaging revealed no lymphadenopathy or organ involvement. A skin biopsy showed increased dermal vascular endothelial cells, significant perivascular lymphocytic infiltration, and interstitial invasion with some lymphocytes entering the epidermis with liquid components and mild spongiosis. There was no notable dyskeratosis. Infiltrating lymphocytes were predominantly CD4-positive and CD8-negative T cells. Initial treatment with fusidic acid ointment was started, but erosion and ulceration worsened, leading to tumor formation and severe pain, making it difficult to open her eyes (Figure 1). A second biopsy was conducted for a definitive diagnosis. A skin biopsy from the left cheek skin tumor revealed dense diffuse infiltration of atypical lymphocytes with nuclear clefting from the dermis to the subcutaneous tissue and into the epidermis (Figures 2A, 2B). The infiltrating lymphocytes were medium-sized with irregular nuclei and predominantly CD4-positive and CD8-negative (Figures 2C-2F).

**Figure 3:**
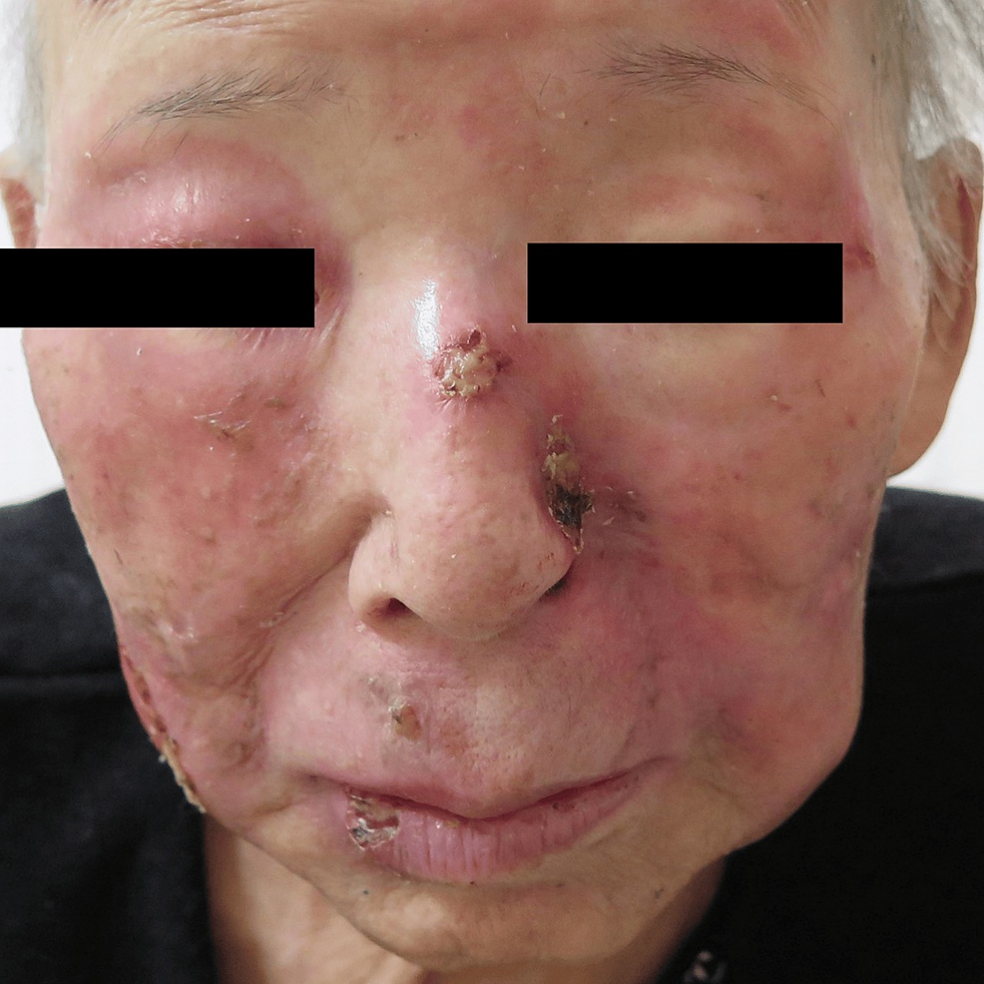
Clinical manifestation The tumors on the face showed rapid size reduction and resolution of erythema by the combination therapy six months after the start of treatment.

Based on these findings, she was diagnosed with MF (4Stage IIB, T2bN0M0B0). Treatment with oral bexarotene (300 mg/m^2^/day) and narrow-band UVB therapy (up to 388 mJ/m^2^ three times a week) was initiated. Although erythema on the chest and back remained stable, tumors persisted on the right eyelid, left cheek, and left sole, with some ulcers worsening. Due to severe impairment and pain caused by the facial tumors, electron beam therapy (total 30 Gr/10 Fr) by Siemens ONCOR Linear Accelerator (Siemens AG, Germany) was administered to the tumors on the face and left sole, leading to rapid size reduction and resolution of erythema (Figure 3). Eye protection with lead lenses was used, and regular ophthalmological observations noted cataracts and superficial punctate keratitis, though no further treatment was required. Post-treatment, hypothyroidism and hyperlipidemia developed possibly due to bexarotene treatment, leading to a reduction in bexarotene dosage and initiation of levothyroxine (100 µg/day) and icosapent ethyl (900 mg three times a day). The final bexarotene dose was adjusted to 225 mg/m^2^/day. Eight months from the initial visit, both tumors and erythema had regressed significantly, with no recurrence of the lesions or organ involvement observed. The treatment continued without any recurrence of the tumor, as confirmed by physical examination and CT 18 months after the treatment commenced. Clinical staging of MF is shown in Table 1.

**Table 1:** Clinical staging of MF * T0 is used for clinical trials in order to track clearance of lesions in the skin compartment. No patient with PCL at time of diagnosis should be T0. † Patients with both erythroderma and tumors may be designated as T4(T3). The BSA of 80% is used to define erythroderma in MF/SS at study entry, but any decrease in BSA during the study does not affect the entry classification. ‡ Abnormal LNs are those now > 1.5 cm LDi according to the Lugano classification and confirmed by imaging. The pathological findings of a representative abnormal LN may apply to all abnormal lymph nodes. § Blood staging for MF/SS is defined currently as B0 = <250/μL of CD4+/CD26− or CD4+/CD7− cells, B1= does not meet criteria for B0 or B2, and B2 = ≥1000/μL of CD4+/CD26−or CD4+/CD7−cells or other aberrant population of lymphocytes identified by flow cytometry. It is expected that patients with high blood tumor burden (B2) will have a clone in the blood that is identical to that in the skin. Nonidentical T-cell clones are often detected in peripheral blood with increasing age and are of unknown clinical significance. MF: Mycosis fungoides; LN: lymph node

Skin (T)	T0*	Absence of clinically suspicious lesions	z	
	T_1_	Patches, plaques, or papules <10% BSA	T_1A_	Patch only lesions
			T_1B_	Plaque/papule^+/−^ patch lesions
	T_2_	Patches, plaques, or papules ≥10% BSA	T_2A_	Patch only lesions
			T_2B_	Plaque/papule^+/−^ patch lesions
					T_3_	One or more tumors ≥1 cm diameter		
	T_4_	Confluence of erythema covering ≥80% BSA†						
	Nodes (N)‡	N_0_	No clinically abnormal LN; no biopsy necessary		
		N_1_	N_1A_	Pathology Dutch grade 1 or NCI LN 0-2: clone negative or equivocal	
N_1B_			Pathology Dutch grade 1 or NCI LN 0-2: clone positive and identical to skin	
N_2_		N_2A_	Dutch grade 2, NCI LN3: clone negative or equivocal	
		N_2B_	Dutch grade 2, NCI LN3: clone positive and identical to skin	
N3‡ (lymphoma)		N_3A_	Dutch grade 3-4, NCI LN4: clone negative or equivocal	
		N_3B_	Dutch grade 3-4, NCI LN4: clone positive and identical to skin	
N_X_		Clinically abnormal peripheral or central lymph node but no pathologic determination of representative LN. Other surrogate means of determining involvement may be determined by Tri-Society consensus		
Viscera (M)	M_0_	No visceral involvement		
	M_1a_	BM only involvement	Clone positive and identical to skin	
			Clone negative or indeterminate	
	M_1b_	Non-BM visceral involvement	Clone positive and identical to skin	
			Clone negative or indeterminate	
	Mx	Visceral involvement is neither confirmed nor refuted by available pathologic or imaging assessment		
Blood (B)§	B_0_	B_0A_	Clone negative or equivocal	Absence of significant blood involvement
		B_0B_	Clone positive and identical to skin	
	B_1_	B_1A_	Clone negative or equivocal	Low blood tumor burden
		B_1B_	Clone positive and identical to skin	
	B_2_	B_2A_	Clone negative or equivocal	High blood tumor burden
		B_2B_	Clone positive and identical to skin	
	Bx	Bx_A_	Clone negative or equivocal	Unable to quantify blood involvement according to agreed-upon guidelines
		Bx_B_	Clone positive and identical to skin	

## Discussion

Cutaneous T-cell lymphoma encompasses a diverse group of non-Hodgkin lymphomas, with MF being the most common subtype. MF manifests in various stages: it appears as patches and plaques in the early stage and progresses to tumors in more advanced forms [3].

MF typically initiates in the patch stage, characterized by eczema-like patches that are frequently misdiagnosed as benign dermatological conditions due to their subtle appearance [4]. MF transitions to the plaque stage reflecting a more active disease state. In its most advanced form, MF progresses to the tumor stage, which is marked by the development of elevated tumors. These tumors have a propensity to ulcerate, serving as a clinical indicator of significant disease advancement and an increased risk of systemic involvement [5].

Diagnostic procedures for cutaneous T-cell lymphoma are critical to its effective management. Early diagnosis primarily relies on skin biopsies, which are essential for histopathological examination [5]. Additional diagnostic tools include blood tests to detect abnormal T-cells, particularly in advanced stages, and imaging techniques such as CT or positron emission tomography scans to assess lymph node involvement and internal organ infiltration [5]. Molecular diagnostics, including T-cell receptor gene rearrangement studies, also play a vital role in confirming the diagnosis [5].

Treatment options for cutaneous T-cell lymphoma vary based on the stage and severity of the disease. Early-stage disease may be effectively managed with topical treatments such as steroids and retinoids, while phototherapy is also commonly used [5]. As the disease progresses, systemic treatments including chemotherapy and biologic therapies become necessary. Advanced stages may require more aggressive treatment strategies, combining several modalities such as radiation therapy, systemic chemotherapy, and newer targeted therapies [5].

Treatment of MF is currently available to use systemic therapies such as retinoids and targeted molecular drugs, as well as local therapies including topical treatments, ultraviolet therapy, and electron beam irradiation. In early systemic therapy, retinoids and interferon-gamma are the first-line options, while targeted molecular drugs and oral histone deacetylase inhibitors such as vorinostat, which is orally administrated as 400 mg, are considered second-line with the efficacy against lymphomas [6,7]. In refractory cases, single-agent or multi-agent chemotherapy and allogeneic hematopoietic stem cell transplantation may be considered. Chemotherapy plays a crucial role in managing advanced MF and Sézary syndrome: however, achieving long-term disease-free survival is challenging. The CHOP regimen shows some effectiveness [8]. In addition, VICOP-B also demonstrates effectiveness for advanced MF [9]. However, the effects of these treatments are limited.

In this case, oral bexarotene, a type of retinoid X receptor agonist, was chosen for treatment [10]. Retinoids are involved in cellular differentiation, proliferation, morphogenesis, and immune function through gene transcription regulation mediated by retinoic acid receptors and retinoid X receptors. Specifically, the retinoid X receptor plays a role in the metabolic control of thyroid hormones, lipids, carbohydrates, and cholesterol, which are considered in the treatment of metabolic diseases and malignancies. Retinoids enter the cell and bind to retinoic acid receptors (RAR) and retinoid X receptors (RXR) [11] which are members of the nuclear receptor family and function within the cell nucleus. RAR and RXR form dimers bind to specific DNA sequences located in the promoter regions of specific genes, thereby regulating gene transcription associated with the physiological and pathological conditions associated with cell differentiation, proliferation, metabolism, and homeostasis [11].

Bexarotene, through the retinoid X receptor, has shown effectiveness in the treatment of MF; however, this is also associated with potential side effects such as hyperlipidemia, neutropenia, and hypothyroidism observed in this case [12]. Thus, additional oral therapies were administered, and the dosage of bexarotene might need to be adjusted.

In this case, oral bexarotene therapy was combined with electron beam irradiation. While narrow-band UVB therapy showed limited efficacy against the skin tumors of MF, possibly due to the deeper infiltration of the tumor cells that are not effectively treated by phototherapy, unlike superficial lesions that can be addressed with this treatment, electron beam irradiation is recommended. This case achieved a favorable clinical outcome with electron beam irradiation to the facial tumors, though risks such as conjunctivitis, cataracts, skin atrophy, and the potential risk for secondary malignancies. Protective usage of lead lenses is useful to prevent cataracts and the occurrence of keratitis. Despite these risks, electron beam irradiation offers the benefit of reducing treatment duration, which might be considered as one of the therapeutic options.

## Conclusions

The oral therapy with bexarotene proved effective despite the risks of side effects like hyperlipidemia. The combination with radiation therapy could reduce the duration to remission and mitigate side effects, making it a valuable option in treatment strategies for tumors of MF on the face. MF, a type of cutaneous T-cell lymphoma, often presents unique challenges due to its manifestation primarily on the skin. The integration of bexarotene with localized radiation offers a promising approach that could revolutionize the management of cutaneous T-cell lymphoma, providing patients with a more effective and potentially faster route to remission. This multimodal strategy underscores the importance of personalized treatment plans that address both the efficacy and QOL for patients with cutaneous T-cell lymphoma.
